# Painful Recall in Elective Electrical Cardioversion with Propofol and the Need for Additional Analgesia

**DOI:** 10.1155/2018/2363062

**Published:** 2018-07-22

**Authors:** D. F. M. van Winden, A. Westra, P. J. W. Dennesen, S. H. J. Monnink, B. C. Verdouw, R. le Kluse

**Affiliations:** ^1^Department of Emergency Medicine, Reinier de Graaf Hospital, Delft, Netherlands; ^2^Department of Intensive Care, Haaglanden Medical Center, The Hague, Netherlands; ^3^Department of Cardiology, Reinier de Graaf Hospital, Delft, Netherlands; ^4^Department of Anaesthesiology, Reinier de Graaf Hospital, Delft, Netherlands

## Abstract

**Introduction:**

Electrical cardioversion (ECV) is a short but painful procedure for treating cardiac dysrhythmias. There is a wide variation regarding the medication strategy to facilitate this procedure. Many different sedative techniques for ECV are described. Currently, the optimal medication strategy to prevent pain in ECV has yet to be established. The role for additional analgesic agents to prevent pain during the procedure remains controversial, and evidence is limited.

**Methods:**

We conducted a prospective multicenter study to determine the incidence of painful recall in ECV with propofol as a sole agent for sedation, in order to assess the indication for additional opioids. In all patients, sedation was induced with propofol titrated till loss of eyelash reflex and nonresponsiveness to stimuli, corresponding to Ramsay Sedation Score level 5-6. ECV was performed with extracardiac biphasic electrical shocks. The primary outcome was painful recall of the procedure, defined as numeric pain rating scale (NRS) ≥ 1. NRS ≥ 4 is considered inadequately treated pain. Secondary outcome parameters were pain at the side of the defipads and muscle pain after ECV.

**Results:**

A total of 232 patients were enrolled in this study. Six patients were excluded due to missing data or violation of study protocol. Three patients reported recall of the procedure, and one patient (0.4%) reported recall of severe pain during the procedure with NRS 7. Two patients (0.9%) reported recall of mild pain with NRS 1–3. Complete amnesia was observed in 223 patients (98.7%), with NRS 0. The mean of the total dose of propofol was 1.1 mg/kg. Fifteen patients (6.6%) experienced pain at the side of the defipads, and six patients (2.7%) complained of muscle pain after the procedure.

**Conclusions:**

In this prospective multicenter study, propofol as a sole agent provided good conditions for ECV with a low incidence of recall. Effective sedation and complete amnesia was achieved in 98.7% of the patients, 0.4% of patients reported recall of severe pain during the procedure, and 0.9% of patients experienced mild pain during the ECV.

## 1. Background

External electrical cardioversion (ECV) is a short and painful procedure for treating cardiac dysrhythmias. There is a wide diversity regarding the medication strategy to facilitate ECV. Cardioversion can be performed using a variety of hypnotic agents, sedative agents, and additional analgesics [1]. The agent of choice is determined by many factors including by the duration of sedation and recovery, the desired depth of sedation, the likelihood of undesirable cardiorespiratory effects, or recall of the procedure.

Propofol is frequently used for ECV because of its ease of titration, rapid onset of action, brief duration of sedation, and amnestic properties [1, 2, 3]. Although propofol has amnestic properties, it is not an analgesic. Previous studies have addressed awareness or painful recall in ECV with propofol. Incidences varied from 0 to 23% [2–14]. These studies had several limitations: recall was not the primary outcome measure, findings were based on small patient groups, results were not differentiated for the sedatives used, supplemental opioids were given, and the studies described a variety of short painful procedures or did not reflect clinical practice.

Currently, the optimal medication strategy to prevent pain in ECV has yet to be established. The evidence for the use of analgesic agents such as opioids in conjunction with sedatives to prevent pain is limited and controversial. The aim of this study was to determine the incidence of painful recall in ECV using propofol as the sole agent for sedation and to assess the need for additional analgesia.

## 2. Methods

### 2.1. Study Design

This was a prospective multicenter study that took place from December 2014 to November 2016 at the Intensive Care Unit (ICU) of Haaglanden Medical Centre and the Cardiac Care Unit (CCU) of Reinier de Graaf Hospital, the Netherlands. In these departments, sedation is performed at the discretion of the attending intensive care specialist or anesthesiologists. Propofol is the sedative agent of choice in ECV. Additional opioids are not given.

The primary outcome was the incidence of painful recall of the ECV after sedation with propofol. Secondary outcomes were pain at the side of the defipads and muscle pain after the procedure.

### 2.2. Patients

All patients (aged 18 years or older) scheduled for cardioversion for arrhythmias such as atrial fibrillation, atrial flutter, or supraventricular tachycardia with an indication for ECV were eligible for study participation. Patients were excluded in case of hypersensitivity to propofol. The regional Medical Ethics Committee Zuidwest Holland, in the Netherlands, exempted this study for formal ethical approval and patient consent. However, verbal consent was asked to all potential study participants before participation in the study. The trial is registered at http://www.trialregister.nl.

### 2.3. Procedure

Depending on the clinical practice of the treating specialist, some patients received a small dose of lidocaine to prevent pain due to injection of propofol. No additional analgesics were given. Anesthesia for ECV was induced with propofol. Propofol was titrated by the attending ICU specialist or anesthesiologist until adequate sedation was achieved. Adequate sedation was defined as a state of unconsciousness with nonresponsiveness to stimuli and loss of eyelash reflex, corresponding with Ramsay Sedation Scale level 5 or 6. In case of inadequate sedation, additional boluses of propofol were given as often as necessary. The dose of additional bolus and the speed of drug injection were not predetermined.

ECV was performed by the cardiologist with extracardiac biphasic electrical shocks ranging from 50 to 200 Joules. If sinus rhythm was not restored, more shocks were delivered. Patients were questioned after the procedure at the moment basal mental status was restored. They were asked to report any pain perception or recall using the Numeric Rating Scale (NRS). The NRS score is a validated score to express the intensity of pain by choosing a number, from 0 (no pain) to 10 (worst pain imaginable). NRS ≥ 4 is used to refer to inadequately treated pain [15]. In this study, we defined NRS 0 as no recall. Ratings of NRS 1–3 corresponded to recall of mild pain, NRS 4-5 to moderate pain, and NRS ≥ 6 to severe pain. Besides painful recall, patients were also asked about local pain during injection of propofol, pain at the side of the defipads and muscle pain after cardioversion.

Records were kept of the pain scores, age, sex, weight, dose of propofol, the use of lidocaine, the number of shocks, and total amount of energy delivered. Data were collected by the ICU specialist, the ICU resident, or the CCU nurse on a preformatted data sheet during the procedure.

### 2.4. Safety

All patients were preoxygenated with 100% oxygen via a nonrebreathing facemask while breathing spontaneously before induction of anesthesia. Noninvasive blood pressures were measured every 3–5 minutes. Oxygen saturation and heart rate were monitored continuously during and after the procedure until patients had returned to their baseline mental status, corresponding with Ramsay Sedation Scale level 2.

In case of apnea, 100% oxygen was administered using bag-valve-mask ventilation. If the patient showed signs of upper airway obstruction, simple airway maneuvers like chin lift and jaw thrust were performed. Adverse events and complications were recorded in the patient files but are not described in this study as they fall beyond the scope of this present study.

### 2.5. Statistical Analysis

As this was a descriptive study, no power calculation was undertaken. Statistical analysis was performed using SPSS Statistics version 17.0. Standard descriptives were obtained. Outcomes in this study included dichotomous outcomes (recall, pain at the side of defipads, and muscle pain), discrete data (NRS and number of shocks), and continuous data (age and propofol dose). Values were expressed as mean ± standard deviation or percentages of patients, as appropriate.

## 3. Results

### 3.1. Patients

A total of 232 patients who underwent ECV facilitated with propofol as the sedating agent were enrolled in the study. Six patients were excluded because of missing data. The mean age was 68.7 years, and 65.5% (148) of the patients were male. The baseline characteristics of the patients are shown in Table 1.

Sixty-two (27.4%) patients received a single dose of lidocaine of 5 to 20 mg to numb the blood vessel before administration of propofol. The mean propofol dose required was 1.1 mg/kg (± 0.3).

Complete amnesia was observed in 223 patients (98.7%) with NRS 0. One patient (0.4%) had reported painful recall of the procedure with NRS 7 and recollection of the two shocks with 150 Joule. This patient received 10 mg lidocaine to numb the blood vessel and 90 mg propofol (0.83 mg/kg). The patient was adequately sedated with loss of eyelash reflex and nonresponsiveness to auditory stimulus. Two patients (0.9%) reported recall of mild pain with NRS 1 and NRS 3, respectively. The patient with NRS 1 received 10 mg lidocaine and 80 mg propofol (0.95 mg/kg), and the patient with NRS 3 received no lidocaine and 90 mg propofol (1.25 mg/kg). In both patients, one shock with 200 Joule was given.

We also studied the need for simple analgesics after ECV. Fifteen patients experienced a burning sensation or pain at the side of the defipads, and six patients complained from muscle pain after the ECV. One, two, or three shocks were needed in respectively 178, 34, and 14 patients. The patients who received 3 shocks reported no pain with NRS 0 after the procedure. Findings of the procedure are presented in Table 2.

## 4. Discussion

### 4.1. Studies Addressing Recall

The aim of this study was to determine the incidence of painful recall in ECV using propofol as the sole agent for sedation. In this multicenter study, painful recall of the ECV was found in 0.4% of the patients.

As far as we know, this is the first study that evaluates the amnesic properties of propofol in ECV as a primary outcome measure in a large group of consecutive patient group.

The previous studies that addressed painful recall after sedation with propofol described incidences of recall varying from 0 to 23% in a variety of short painful procedures [2–14]. These studies have several limitations. For example, Swann et al. [4] described 7.4% recall in 41 cardioversions and 84 orthopaedic reductions with a wide range of drug combinations used. Kaye and Govier [2] and Valtonen et al. [11] described complete amnesia of ECV in all patients sedated with propofol. However, in none of these studies recall was the primary outcome. The presence of recall was not routinely noted, which can result in an underreporting of the incidence of recall. Furthermore, Valtonen et al. [11] sedated 15 patients undergoing ECV with a mean dose of 2.5 mg/kg propofol. This dosage does not reflect current clinical practice. Miner et al. [10] described 16.5% recall of painful procedures after procedural sedation with propofol or propofol and alfentanil. However, in this RCT, only two patients underwent cardioversion, out of the 150 included patients. Furthermore, in most studies addressing recall, supplemental opioid was added to propofol sedation [3, 5, 6, 8, 10, 12–14].

The evidence for the use of opioids in conjunction with propofol to prevent pain is limited and controversial. The studies that added opioids to propofol in short painful procedures showed similar incidences of recall compared to studies in which procedures were performed with propofol as a sole agent [2–14]. Miner et al. stated that the combination of propofol with remifentanil has demonstrated to act synergistic in controlling a response in painful procedures [16]. In another study of Miner et al., stress markers were obtained in the emergency department after short painful procedures were done in 20 patients sedated with propofol as a sole agent or propofol and alfentanil. The addition of alfentanil to propofol did not seem to have an effect on serum catecholamine as marker of physiologic stress in [17].

Moreover, it can be debated whether pain experienced during a procedure, without recalling it afterwards, is clinically relevant [18].

Furthermore, studies have demonstrated that administering opioids in addition to propofol may even contribute to increased rates of respiratory depression and clinical interventions [8, 10, 12–14, 19–22]. However, also propofol as a sole agent may cause dose‐dependent adverse events such as respiratory depression and hemodynamic compromise. The safety and adverse events of propofol have been studied widely in the past and were beyond the scope of this study. However, no adverse events were reported in our study population, although this might have been underreported since it was not a primary endpoint for this study.

In our study, complete amnesia of the ECV was observed in 98.7% of the patients sedated with propofol, and 0.9% did recall a feeling of mild pain (NRS 1–3) during the procedure. Previous literature on the Numeric Rating Scale determined NRS ≥ 4 as inadequate treated pain [15]. In this study 0.4% of patients experienced such severe pain (NRS 7). It remains an issue of discussion to what extent any incidence of recall is acceptable in clinical practice. Based on the literature and our findings, we do not recommend additional opioids to propofol sedation in ECV. In the previous literature, opioids have not proven to be beneficial in ECV. On the contrary, opioid combined with propofol can increase the likelihood of adverse events [9, 10]. However, these studies did not have recall as primary outcome. A randomized controlled trial that compares recall in ECV with propofol in one arm and propofol and an opioid in the other arm would be recommended to further address this subject.

The secondary outcomes of this study were pain at the time of propofol injection and pain at the site of the defipads. The literature describes injection pain in 2–33% of the patients [3, 7, 22]. The administration of intravenous lidocaine is found to prevent discomfort with 60% of the time with a rubber tourniquet in place 30 to 120 seconds before propofol administration [23]. In our study, 5.8% of the patients experienced pain at the time of propofol injection. Three of these patients (1.3%) who received lidocaine prior to propofol injection experienced injection pain. Muscle pain, burning sensations, or pain at the side of the defipads was reported by 7.3% of the patients.

### 4.2. Study Design and Limitations

The variance to the dosage of propofol can be seen as a limitation of this study. However, our aim was to determine the incidence of painful recall in propofol sedation in current clinical practice. In current clinical practice, careful titration of propofol until adequate sedation is achieved is preferred above the large boluses. Titration of propofol is known to prevent profound hypotension and oxygen desaturation [1, 3, 5, 11]. In contrast to previous studies that reported recall, the doses of propofol in this study appear relatively ‘light' for the patients' weight (1.1 mg/kg).

Conversely, the dosage of propofol in this study can also be called rather ‘high' for this study population with a mean age of 68.7. In the elderly population, 1 mg/kg propofol is generally recommended to induce general anesthesia. This relative overdose of propofol might have contributed to the low incidence of reported recall.

Furthermore, speed of drug injection and time between additional boluses can be a confounding factor since this was not standardized. In this cardiologic population, slow drug distribution causes a longer time to achieve adequate sedation. Also, additional boluses of propofol were not predetermined. This might have led to high doses of propofol in some patients depending on the personal preference of the attending specialist. Nevertheless, these unpredictable factors contribute to the aim of our study, to evaluate recall in current clinical practice. Variety is part of clinical practice and despite that variety propofol proves to be providing sufficient sedation to prevent recall of the ECV.

Another limitation of this study is the missing data regarding the amount of electricity delivered to achieve cardioversion. Although the Joules used in the patients with recall are known, data is missing for 20/223 patients without recall. It is possible that the height of energy might be related to a painful recall, but given the small number of patients with recall, a subset analysis of these patients with regard to this factor will have minimal impact on the study outcomes.

## 5. Conclusion

In this prospective multicenter study, propofol as a sole agent provided good conditions for ECV with a low incidence of recall. Effective sedation and complete amnesia were achieved in 98.7% of the patients. 0.4% of patients reported severe painful recall of the procedure and 0.9% of patients experienced mild pain during the ECV.

## Figures and Tables

**Table 1:** Patient Characteristics of the study subjects and doses of propofol and analgesic agents.

Age (years), median (range)	68 (30–93)
Weight (kg), mean (±SD)	86.5 ± 17.7
Length (cm), mean (±SD)	177.9 ± 9.9
Lean body mass (kg), mean (±SD)	61.6 ± 12.4
Gender (male/female) %	148/78 (65.5/34.5)
Total dose of propofol (mg), mean (±SD)	90.8 ± 37.6
Dose of propofol/weight (mg/kg), mean (±SD)	1.1 ± 0.3
Dose of propofol/lean body weight (mg/kg), mean (±SD)	1.5 ± 0.7
Number of patients given lidocaine (*n*, %)	62 (27.4)

**Table 2:** Details of findings of the ECV.

Number of shocks (*n*/*N*) (%)	
1	178 (76.7)
2	34 (15.0)
3	14 (6.2)
Reported amnesia NRS 0	223 (98.7)
Reported painful recall (NRS 1–3) of the procedure (*n*/*N*) (%)	2 (0.9)
Reported painful recall (NRS ≥ 4) of the procedure (*n*/*N*) (%)	1 (0.4)
Reported pain at the time of propofol injection (*n*/*N*) (%)	13 (5.8)
Reported pain at the time of injection when used lidocaine (*n*/*N*) (%)	3 (1.3)
Reported pain at the site of the defipads after the procedure (*n*/*N*) (%)	15 (6.6)
Reported muscle pain after the procedure (*n*/*N*) (%)	6 (2.7)

## Data Availability

The data used to support the findings of this study are available from the corresponding author upon request.
